# Bullous Parametric Response Map for Functional Localization of COPD

**DOI:** 10.1007/s10278-021-00561-z

**Published:** 2022-01-11

**Authors:** Kuo-Lung Lor, Yeun-Chung Chang, Chong-Jen Yu, Cheng-Yi Wang, Chung-Ming Chen, On behalf of the TCORE consortium

**Affiliations:** 1 Department of Biomedical Engineering, National Taiwan University, Taipei, Taiwan; 2 Department of Medical Imaging, National Taiwan University Hospital and College of Medicine, National Taiwan University, Taipei, Taiwan; 3 Department of Internal Medicine, National Taiwan University Hospital and College of Medicine, National Taiwan University, Taipei, Taiwan; 4 Department of Internal Medicine, College of Medicine, Cardinal Tien Hospital and School of Medicine, Fu-Jen Catholic University, New Taipei City, Taiwan

**Keywords:** BLVR, PRM, PFT, Predictive model, Functional localization

## Abstract

Advanced bronchoscopic lung volume reduction treatment (BLVR) is now a routine care option for treating patients with severe emphysema. Patterns of low attenuation clusters indicating emphysema and functional small airway disease (fSAD) on paired CT, which may provide additional insights to the target selection of the segmental or subsegmental lobe of the treatments, require further investigation. The low attenuation clusters (LACS) were segmented to identify the scalar and spatial distribution of the lung destructions, in terms of 10 fractions scales of low attenuation density (LAD) located in upper lobes and lower lobes. The LACs of functional small airway disease (fSAD) were delineated by applying the technique of parametric response map (PRM) on the co-registered CT image data. Both emphysematous LACs of inspiratory CT and fSAD LACs on expiratory CT were used to derive the coefficients of the predictive model for estimating the airflow limitation. The voxel-wise severity is then predicted using the regional LACs on the co-registered CT to indicate the functional localization, namely, the bullous parametric response map (BPRM). A total of 100 subjects, 88 patients with mild to very severe COPD and 12 control participants with normal lung functions (FEV_1_/FVC % > 70%), were evaluated. Pearson’s correlations between FEV_1_/FVC% and LAV%_HU-950_ of severe emphysema are − 0.55 comparing to − 0.67 and − 0.62 of LAV%_HU-856_ of air-trapping and LAV%_fSAD_ respectively. Pearson’s correlation between FEV_1_/FVC% and FEV_1_/FVC% predicted by the proposed model using LAD% of HU-950 and fSAD on BPRM is 0.82 (*p* < 0.01). The result of the Bullous Parametric Response Map (BPRM) is capable of identifying the less functional area of the lung, where the BLVR treatment is aimed at removing from a hyperinflated area of emphysematous regions.

## Introduction

COPD is a progressive, irreversible disease characterized by alveolar destructions due to small airways disease and loss of supporting tissue due to emphysema. Currently, the treatment options for patients suffering from emphysema are limited. Various techniques of bronchoscopic lung volume reduction treatment (BLVR) are recommended by the international expert panel as a minimally invasive procedure for improving pulmonary function, exercise capacity, and life quality in patients with COPD. These techniques include valves, coils, vapor thermal ablation, and sealant. Each method requires careful evaluation due to its limitation in treatment. In particular, endobronchial valves (EBVs) improve lung volumes and allow for the expansion of lung tissue. However, this lobar exclusion cannot enroll patients who have extensive interlobar collaterals in the emphysematous lung. On the other hand, endobronchial coil therapy which is independent of collateral ventilation improves the elastic recoil by the compression and redistribution of airflow toward healthier segments. Bronchoscopic thermal vapor ablation (BTVA, Uptake Medical Corporation, Seattle, WA, USA) reduces lung volume by the instillation of heated water in the most diseased segments to provoke irreversible parenchymal fibrosis and scarring of emphysematous tissue [1, 2]. The challenge for the treatment success is, nevertheless, to define the specific signatures of the “most diseased segments or subsegments of the lobe”[3].

Quantitative computed tomography (QCT) can sensitively assess the size and spatial distribution of low attenuations clusters (LACs) as the emphysematous characteristics in patients with chronic obstructive pulmonary disease (COPD). The radiographic assessment has been developed for patient inclusion using quantitative computed tomography (QCT) as the predictors of treatment outcome, such as fissure integrity for evaluating the collateral ventilation and low attenuation clusters for evaluating emphysema heterogeneity. In the current paradigm of BLVR-specific quantitative CT imaging, emphysema lesion is segmented by the threshold of -950 Hounsfield units (HU) to extract low attenuation clusters (LACs) in the inspiratory CT data. Emphysema severity is then computed from the low attenuation volume percentage (LAV%) as the ratio of LACs to whole lung volume. However, there is no consensus definition for the emphysema heterogeneity score (HS), and many studies have proposed methods using interlobar LAV% [3, 4]. Recently, Lor et al. have developed a LAC-based representation of emphysematous lesions grouped by four proportional scales in upper and lower lobes. The compositions of categorized LACs are used to build the predictive modeling of voxel-wise airflow limitation. As a result, instead of most “emphysema-destroyed lobe,” the most “functionally affected” tissue can be identified for the treatment [5].

Although previous studies mainly take emphysema heterogeneity on inspiratory CT into consideration, BLVR is expected to treat the lobe with hyperinflation, which results from gas being trapped during the expiratory phase of the breathing. As suggested in the findings of recent ex vivo studies using high-resolution microcomputed tomography (microCT), the destruction of the terminal bronchiole is hypothetically the primary site in the lungs of patients with mild and moderate COPD, but with no sign of emphysema [6–8]. On the other hand, other studies have shown cases of emphysema without obstruction on spirometry [9]. The discordance between the results of the pulmonary function test (PFT) and structural alterations in emphysema and functional small airway disease only highlights partial aspects of the disease complexity. While the PFT provides a global assessment to diagnose COPD, CT imaging quantifies local structural and functional abnormalities, which induce the heterogeneity of COPD phenotypes, and further increase the disease complexity. As a result, both emphysema and small airway disease should be assessed for the COPD heterogeneity in the target lobe selection.

Emphysema can be obtained from LACs on inspiratory CT, whereas the morphology of the small airway (< 2 mm in diameter) is below the resolution of CT image data. Well-accepted quantification of emphysema severity is the percentage of LAV segmented by the threshold of -900 ~ -950 Hounsfield units (HU) in total lung volume on inspiratory CT, whereas LAV% segmented by a threshold of -856 HU on expiratory CT has been used as a measure of air-trapping due to the functional small airway disease (fSAD). While both emphysema and fSAD are the main components of the heterogeneous COPD process, the surrogate measure of air-trapping is partially overlapped with emphysematous destruction. To quantify the COPD phenotypes, Galbán et al. co-registered inspiratory and expiratory CT scans to distinguish the relative contributions of fSAD and emphysema for a more accurate diagnosis [10]. Here, we co-registered the paired inspiratory and expiratory CT to identify the emphysema LACs as well as the fSAD LACs. After deriving the prediction model for the airflow limitation, the bullous parametric response map (PRM) (BPRM) is then constructed by accumulating the functional contributions of the regional low attenuation densities (LADs).

## Materials and Methods

### Patient and Public Involvement

The volumetric CT scans were taken at full inspiration and full expiration for each subject. However, the interpretation and conclusions contained in this study are those of the authors alone. A total of 88 subjects with symptoms of chronic obstructive pulmonary disease (GOLD stage 1 ~ 4) and 12 normal subjects was included. Subject demographics are summarized in Table 1. All subjects are evaluated in the correlational studies, and 80 of randomly selected subjects are trained in the predictive modeling for testing on the remaining 20 subjects as the setup for repeated fivefold cross-validations.

**Table 1:** Subject demographics (*n* = 100)

Parameter	Mean (± std) or count (%)
Sex male	98
Age	67.87 (9.63)
Height (cm)	166.88 (6.78)
Weight (kg)	67.22 (12.94)
BMI	24.05 (4.0)
FEV_1_/FVC %	54.55 (12.96)
FEV_1_% predicted	66.53 (22.29)

*BMI* body mass index, *FEV*_*1*_ forced expiratory volume in one second, *FVC* functional vital capacity

### CT Image Analysis and PRM

The co-registration of expiratory and inspiratory was performed in three main steps: lung mask segmentation, three-dimensional non-rigid point set registration, and image deformation. CT Automated airway and lung segmentation were performed using in-house software. In particular, the method of lung segmentation affects the accuracy of LAV%. The lung mask was segmented by applying the adaptive region growing with the upper bound value of HU 200 and using the airway mask as the starting point. After all the dark regions (all voxels with HU less than 200) were segmented and marked as the initial lung mask, the enclosed vascular regions were included by morphological operations (such as closing and filling by dilation and erosion). The separation of left and right lobes was performed by erosion to detach the anterior borders of the lobes. Then, the completed left and right lobes were obtained by the dilation separately.

To minimize the contribution of the airway lumen, the airway was removed from the lung mask. Once the lung masks were extracted from the paired CT dataset, the registration of expiratory CT (floating) aligning with inspiratory CT (reference) takes place in the left and right lobes separately. The evenly distributed sparse points of the lung mask were used as the input of the registration algorithm. We applied the coherent point drift (CPD) to transform the floating-point set to the reference point set. The outstanding performance of CPD was evaluated thoroughly in the work of Wang et al. [11] using COPDgene dataset of DIR-lab (http://www.dir-lab.com/index.html) [12]. While preserving the shape context, CPD also transforms the spatial distribution of the interior point set within the lung mask. The transformed interior-point set can be used as the landmarks for the image deformation, where the co-registered volume data is the result of interpolation within landmarks.

To furtherly investigate the impact of the regional emphysema on lung functions, not only the lobes are labeled as left and right lobes, but also the upper and lower lobes. The upper lobes include the right upper lobe and the left upper lobe, while the lower lobes are the right middle lobe, the right lower lobe, and the left lower lobe. The method of segmenting the upper and lower lobes is derived from the fissures as the results of thin plate structures of the hessian filter. The sample points of the fissure are the control points of thin plate spline, TPS, which can be used to delineate the borders between lobes.

### Measurements of Low Attenuation Volume Percentage

The low attenuation volume is the targeting voxel of the binarized image using a fixed HU threshold value on CT. LAV% is the percentage of targeting voxels in the whole lung. In this study, we include four types of targeting voxels: mild emphysema on inspiratory CT using the threshold of HU-920, severe emphysema on inspiratory CT using the threshold of HU-950, air-trapping on expiratory CT using the threshold of HU-856, and fSAD of PRM which is a result of air-trapping on co-registered expiratory excluding overlapped emphysema voxels on inspiratory. The notations are LAV%_Emph920_, LAV%_Emph950_, LAV%_AirT_, and LAV%_fSAD_, respectively.

### Modeling of the Low Attenuation Cluster using the Local Maxima of Bulla Voxels

This study utilized an algorithm that was developed in the previous work to find the LACs in the binarized image using a fixed HU threshold value on inspiratory CT and expiratory CT. By applying iterative erosion to the binarized image data, each LAC will accumulate the number of eroded voxels from previous steps of erosion. The method of obtaining LACs is described in the works of Lor et al. [5]. The fraction density of LACs or low attenuation density (LAD) is the total number of bulla voxels divided by the total number of parenchymal voxels. The summation of LADs is the same as the low attenuation volume percentage (LAV%). The corresponding LAD notations for each type of targeting voxel are LAD%_Emph920_, LAD%_Emph950_, LAD%_AirT_, and LAD%_fSAD_. While LACs of emphysema and air-trapping are segmented directly from binarized image using single-value thresholding, LACs of fSAD are not based on the fSAD of PRM. The excluding emphysematous voxels (HU < -950 on both inspiratory CT and co-registered expiratory CT) destroy the completeness of bullous structures and create more fragments than the number of visually observed clusters. The shortcoming of extracting LACs in the PRM approach can be registration algorithm dependent and thus lowers the reproducibility. More accountable approaches are referring to the classification based on the predominant ratio between emphysematous voxels (HU-950) and air-trapping voxels (HU-856). The emphysematous predominant LACs are excluded from co-registered expiratory CT, whereas the LAD%_fSAD_ of LAC_fSAD_ is the percentage of fSAD voxels determined by PRM.

### Statistical Analysis

Pearson’s test was performed to report the correlations between LAV% and FEV_1_% predicted and FEV1/FVC% on inspiratory CT and expiratory CT. The cluster analysis of LADs is performed by ckmeans.1d.dp method (which is based on dynamic programming for optimal one-dimension K-means clustering) to cluster into 10 size ranges [13]. A total of 5.3 million LACs from all the datasets on inspiratory CT and 2.8 million LACs from expiratory CT were collected to illustrate their relationship with lung functions.

The initial correlation study is conducted to FEV_1_/FVC% as the predicting target. Although the GOLD classification proposes a COPD grading system, the categorical staging result has a weak correlation with LAV%. In this study, the full dataset was evaluated for the correlation study of the disease, and the predictive model derived from the training dataset will be used to predict the outcome of the testing dataset. The final model was tested with repeated fivefold cross-validation. The full model of multivariable linear regression associated with FEV_1_/FVC% was tested for CT emphysema and air-trapping using 40 possible predictor variables. We then used backward selection to reduce the collinearity and use only 16 predictors in the final model. The final model has *p*-value of less than 0.05 in each predictor. All statistical analyses were performed in R statistical software (version 3.6.1; R Foundation for Statistical Computing, Vienna, Austria).

## Results

### Subject Characteristics

**Fig. 1:**
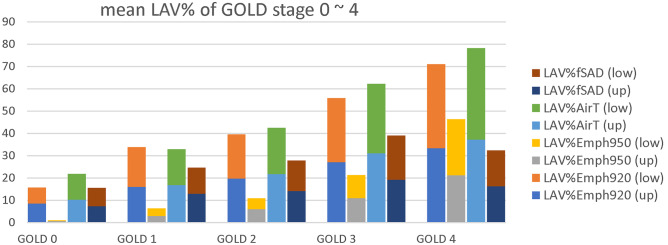
Distribution of LAV% at a different stage of GOLD (*n* = 100); low is the measurements of lower lobes and up is the measurements of upper lobes

The study included 100 participants with inspiratory and expiratory CT taken at the same time before the treatment. The dataset has shown the normal distribution of lung function. The regions of the whole lung are partitioned into upper and lower lobes. In contrast to the conventional emphysema threshold of HU-950, the proposed method uses single HU-930 to segment the mild emphysematous regions [14]. The baseline characteristics of participants are shown according to GOLD stages 0 through 4 in Table 2. Both the mean values of LAV%_Emph_ and LAV%_AirT_ are increased relative to the COPD severity graded by GOLD as expected. There is no significant difference in LAV% between upper lobes and lower lobes at each stage. While FEV_1_% predicted is the GOLD criteria for staging severity (stage 1, mild: FEV_1_% predicted ≥ 80%; 2, moderate: 50 ~ 70%; 3, severe: 30 ~ 49%; 4, very severe: < 30%), the mean values of LAV% at each stage increase accordingly in the categorical subjects as shown in Fig. 1. BMI declines gradually as the COPD gets more severe.

**Table 2:** Subject demographics based on GOLD categories

Parameter	Mean (± std) or count (%)				
GOLD	**0 (normal)**	**1 (mild)**	**2 (moderate)**	**3 (severe)**	**4 (very severe)**
Subjects	12	16	48	22	2
Sex male	11	16	47	22	2
Age	61.67 (10.41)	68.12 (11.82)	69.69 (8.88)	67.14 (8.43)	67.50 (7.78)
LAV%_Emph920_ (up)	8.49 (6.46)	16.01 (6.72)	19.76 (9.54)	26.98 (6.91)	33.25 (0.62)
LAV%_Emph920_ (low)	7.18 (5.05)	17.79 (9.24)	19.76 (8.46)	28.91 (6.85)	37.78 (7.14)
LAV%_Emph920_	15.67 (11.33)	33.80 (15.34)	39.52 (16.67)	55.89 (12.80)	71.03 (7.76)
LAV%_Emph950_ (up)	0.57 (0.81)	2.92 (3.03)	5.99 (6.44)	10.94 (6.47)	21.12 (2.51)
LAV%_Emph950_ (low)	0.40 (0.55)	3.43 (3.15)	4.92 (4.04)	10.38 (5.13)	25.29 (10.70)
LAV%_Emph950_	0.97 (1.33)	6.35 (5.83)	10.92 (9.76)	21.33 (10.98)	46.41 (8.20)
LAV%_AirT_ (up)	10.18 (11.97)	16.82 (10.17)	21.68 (10.44)	31.08 (7.84)	37.18 (0.01)
LAV%_AirT_ (low)	11.68 (12.70)	16.08 (9.92)	20.80 (9.30)	31.13 (8.50)	41.08 (4.40)
LAV%_AirT_	21.87 (24.61)	32.89 (19.55)	42.48 (18.38)	62.16 (15.23)	78.26 (4.41)
LAV%_fSAD_ (up)	7.29 (9.42)	12.95 (8.31)	14.07 (7.03)	19.22 (6.62)	16.25 (5.33)
LAV%_fSAD_ (low)	8.24 (9.64)	11.66 (7.71)	13.81 (7.11)	19.85 (6.40)	16.16 (4.40)
LAV%_fSAD_	15.53 (18.98)	23.62 (15.80)	27.88 (13.43)	39.07 (12.48)	32.41 (0.93)
Height (cm)	164.73 (6.67)	169.19 (7.41)	167.20 (7.04)	165.99 (5.62)	163.50 (7.78)
Weight (kg)	69.58 (14.12)	70.41 (11.69)	68.32 (13.65)	61.98 (10.45)	56.00 (0.00)
BMI	25.56 (4.50)	24.51 (2.99)	24.37 (4.28)	22.45 (3.46)	21.02 (1.99)
FEV_1_/FVC %	74.80 (3.41)	63.45 (5.61)	54.53 (7.32)	39.31 (6.82)	30.01 (4.38)
FEV_1_% predicted	95.98 (18.23)	92.56 (8.55)	64.56 (7.90)	39.74 (5.33)	23.62 (0.86)

*FEV*_*1*_ forced expiratory volume in one second, *FVC* functional vital capacity, *GOLD* the global initiative for chronic obstructive pulmonary disease, *BMI* body mass index

### Correlation Study Between Lung Functions and LAV%

**Fig. 2:**
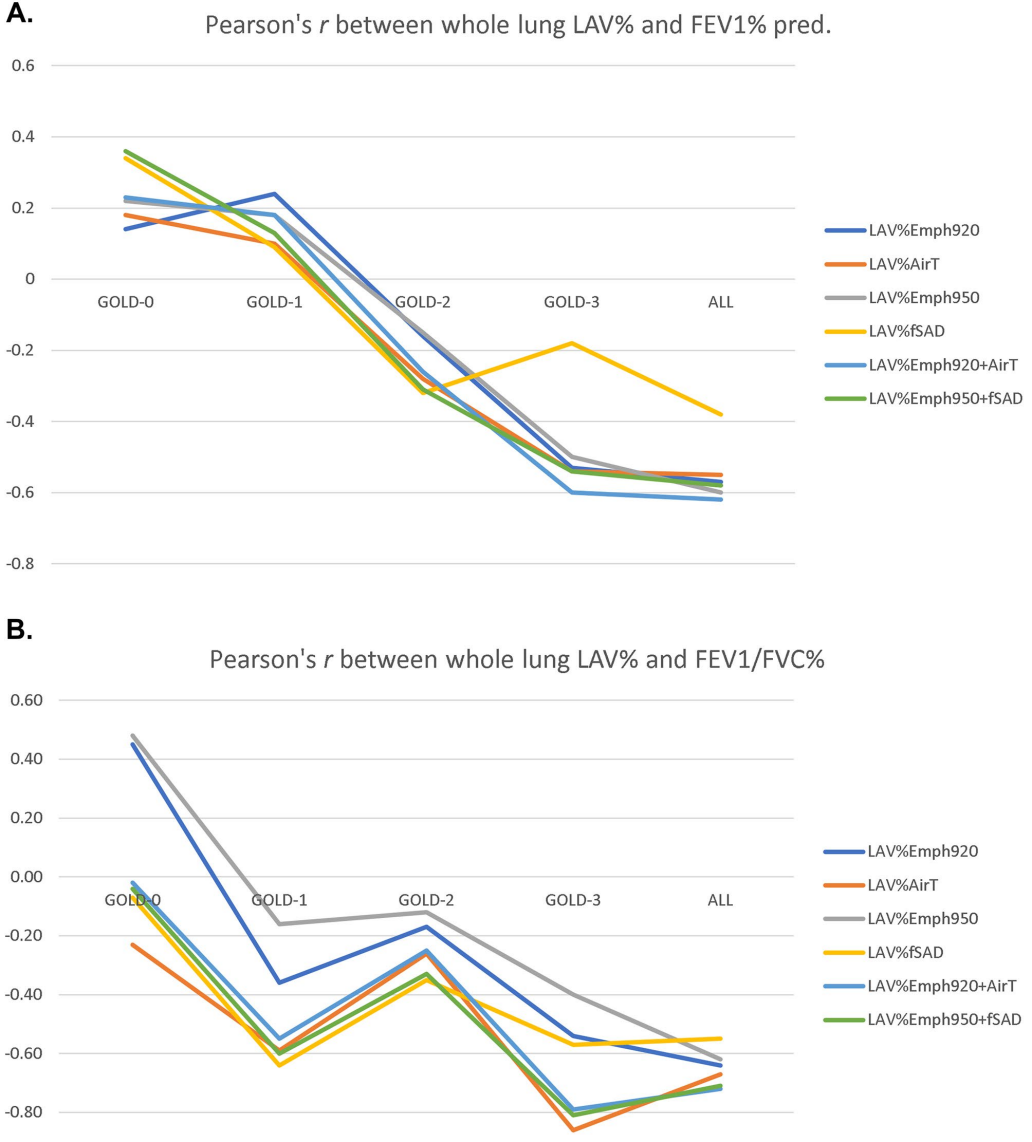
Pearson’s correlation between whole lung LAV% and lung function at different GOLD stage. The results of the plot **A** and **B** are based on the measurement given in Table 3. Plot **A** compares the average LAV% with FEV1% pred, whereas plot **B** compares with FEV1/FVC%

In the past decades, Pearson’s correlations between interlobar LAV% and PFT have been studied extensively, and Spearman’s rank analysis was used to assess the categorical correlation between interlobar LAV% and GOLD stage classification [15]. In contrast to previous studies, we sought to characterize the heterogeneity of COPD phenotypes in terms of contribution at different stages of development. Figure 2 shows correlation coefficients between LAV% and PFT at the different GOLD stages. It can be seen that although the total dataset (*n* = 100) has significant correlation with PFT (*r* >|0.5|, *p* < 0.01), only patients at GOLD stage 3 (*n* = 22) have contributions with significant correlation (*r* >|0.5|, *p* < 0.05) with FEV_1_% pred. Table 3 shows correlation coefficients between various LAV% and PFT at the different GOLD stages. In contrast to the results of FEV_1_% pred, it can be seen that while severe emphysema (GOLD 3) has the most contribution to FEV_1_/FVC% in lung function decline, air-trapping and other composite measurements using both inspiratory and expiratory CT have a better linear association with earlier stages of COPD. Interestingly, the weaker correlation at stage 2 (*r* <|0.3|), as comparing to stage 1 and 3 (*r* >|0.5| and *r* >|0.8|), suggested a non-linear relationship between LAV% and FEV1/FVC% in patients with the mild syndrome. The resulting correlation comparison of Table 3 is shown in Fig. 2. While the ideal Pearson’s correlation coefficients are expected to be equally strong in all GOLD stages, FEV_1_% predicted only has a moderate correlation with severe COPD, and FEV_1_/FVC% was shown stronger in all stages as compared to FEV_1_% precited.

**Table 3:** Pearson’s correlation between LAV% and lung function: *r* (*p*-value)

GOLD Lobes vs PFT			0 (*n* = 12)	1 (*n* = 16)	2 (*n* = 48)	3 (*n* = 22)	4^a^ (*n* = 2)	All (*n* = 100)
LAV%_Emph920_	Upper	FEV_1_% pred	0.15 (0.65)	0.37 (0.15)	− 0.16 (0.29)	− 0.48 (0.02)	N/A	− 0.52 (< 0.01)
		FEV_1_/FVC %	0.39 (0.21)	− 0.21 (0.41)	− 0.16 (0.27)	− 0.42 (0.05)	N/A	− 0.58 (< 0.01)
	Lower	FEV_1_% pred	0.13 (0.67)	0.13 (0.63)	− 0.14 (0.36)	− 0.50 (0.02)	N/A	− 0.56 (< 0.01)
		FEV_1_/FVC %	0.50 (0.09)	− 0.44 (0.08)	− 0.15 (0.31)	− 0.58 (< 0.01)	N/A	− 0.65 (< 0.01)
	Whole	FEV_1_% pred	0.14 (0.65)	0.24 (0.37)	− 0.16 (0.28)	− 0.53 (0.01)	N/A	− 0.57 (< 0.01)
		FEV_1_/FVC %	0.45 (0.14)	− 0.36 (0.17)	− 0.17 (0.25)	− 0.54 (< 0.01)	N/A	− 0.64 (< 0.01)
LAV%_AirT_	Upper	FEV_1_% pred	0.17 (0.58)	0.19 (0.48)	− 0.25 (0.08)	− 0.51 (0.01)	N/A	− 0.52 (< 0.01)
		FEV_1_/FVC %	− 0.20 (0.53)	− 0.44 (0.08)	− 0.26 (0.08)	− 0.84 (< 0.01)	N/A	− 0.64 (< 0.01)
	Lower	FEV_1_% pred	0.18 (0.57)	0.0 (0.97)	− 0.28 (0.05)	− 0.52 (0.01)	N/A	− 0.55 (< 0.01)
		FEV_1_/FVC %	− 0.26 (0.41)	− 0.71 (< 0.01)	− 0.23 (0.1)	− 0.80 (< 0.01)	N/A	− 0.66 (< 0.01)
	Whole	FEV_1_% pred	0.18 (0.57)	0.10 (0.70)	− 0.28 (0.04)	− 0.54 (< 0.01)	N/A	− 0.55 (< 0.01)
		FEV_1_/FVC %	− 0.23 (0.47)	− 0.59 (0.01)	− 0.26 (0.07)	− 0.86 (< 0.01)	N/A	− 0.67 (< 0.01)
LAV%_Emph950_	Upper	FEV_1_% pred	0.22 (0.48)	0.26 (0.33)	− 0.14 (0.33)	− 0.52 (< 0.01)	N/A	− 0.54 (< 0.01)
		FEV_1_/FVC %	0.50 (< 0.1)	0.05 (0.86)	− 0.19 (0.19)	− 0.35 (0.11)	N/A	− 0.56 (< 0.01)
	Lower	FEV_1_% pred	0.20 (0.52)	0.09 (0.74)	− 0.14 (0.35)	− 0.42 (0.06)	N/A	− 0.60 (< 0.01)
		FEV_1_/FVC %	0.43 (0.16)	− 0.35 (0.19)	0.0 (0.98)	− 0.41 (0.05)	N/A	− 0.62 (< 0.01)
	Whole	FEV_1_% pred	0.22 (0.49)	0.18 (0.50)	− 0.15 (0.31)	− 0.50 (< 0.05)	N/A	− 0.60 (< 0.01)
		FEV_1_/FVC %	0.48 (0.11)	− 0.16 (0.54)	− 0.12 (0.40)	− 0.40 (0.06)	N/A	− 0.62 (< 0.01)
LAV%_fSAD_	Upper	FEV_1_% pred	0.33 (0.29)	0.13 (0.61)	− 0.27 (0.06)	− 0.05 (0.81)	N/A	− 0.34 (< 0.01)
		FEV_1_/FVC %	− 0.03 (0.92)	− 0.53 (< 0.05)	− 0.27 (< 0.05)	− 0.47 (< 0.05)	N/A	− 0.50 (< 0.01)
	Lower	FEV_1_% pred	0.35 (0.27)	0.03 (0.90)	− 0.33 (< 0.05)	− 0.30 (0.17)	N/A	− 0.39 (< 0.01)
		FEV_1_/FVC %	− 0.10 (0.73)	− 0.73 (< 0.01)	− 0.39 (< 0.01)	− 0.61 (< 0.01)	N/A	− 0.56 (< 0.01)
	Whole	FEV_1_% pred	0.34 (0.27)	0.09 (0.75)	− 0.32 (< 0.05)	− 0.18 (< 0.42)	N/A	− 0.38 (< 0.01)
		FEV_1_/FVC %	− 0.07 (0.82)	− 0.64 (< 0.01)	− 0.35 (< 0.01)	− 0.57 (< 0.01)	N/A	− 0.55 (< 0.01)
LAV%_Emph920+AirT_	Upper	FEV_1_% pred	0.24 (0.45)	0.30 (0.25)	− 0.23 (0.12)	− 0.56 (< 0.01)	N/A	− 0.57 (< 0.01)
		FEV_1_/FVC %	0.00 (0.98)	− 0.40 (0.11)	− 0.23 (0.11)	− 0.73 (< 0.01)	N/A	− 0.67 (< 0.01)
	Lower	FEV_1_% pred	0.22(0.49)	0.07 (0.79)	− 0.26 (0.08)	− 0.56 (< 0.01)	N/A	− 0.62 (< 0.01)
		FEV_1_/FVC %	− 0.05 (0.86)	− 0.64 (< 0.01)	− 0.23 (0.05)	− 0.76 (< 0.01)	N/A	− 0.73 (< 0.01)
	Whole	FEV_1_% pred	0.23 (0.47)	0.18 (0.49)	− 0.26 (0.07)	− 0.60 (< 0.01)	N/A	− 0.62 (< 0.01)
		FEV_1_/FVC %	− 0.02 (0.94)	− 0.55 (0.02)	− 0.25 (0.08)	− 0.79 (< 0.01)	N/A	− 0.72 (< 0.01)
LAV%_Emph950+fSAD_	Upper	FEV_1_% pred	0.36 (0.26)	0.21 (0.44)	− 0.27 (0.06)	− 0.52 (< 0.01)	N/A	− 0.55 (< 0.01)
		FEV_1_/FVC %	0.01 (0.97)	− 0.47 (0.07)	− 0.30 (< 0.05)	− 0.75 (< 0.01)	N/A	− 0.67 (< 0.01)
	Lower	FEV_1_% pred	0.36 (0.25)	0.05 (0.84)	− 0.32 (< 0.05)	− 0.51 (< 0.01)	N/A	− 0.58 (< 0.01)
		FEV_1_/FVC %	− 0.08 (0.79)	− 0.69 (< 0.01)	− 0.31 (< 0.05)	− 0.78 (< 0.01)	N/A	− 0.71 (< 0.01)
	Whole	FEV_1_% pred	0.36 (0.25)	0.13 (0.62)	− 0.31 (0.05)	− 0.54 (< 0.01)	N/A	− 0.58 (< 0.01)
		FEV_1_/FVC %	− 0.04 (0.90)	− 0.60 (< 0.01)	− 0.33 (< 0.05)	− 0.81 (< 0.01)	N/A	− 0.71 (< 0.01)

*PFT* pulmonary function test, *LAV* low attenuation volume, *FEV1* forced expiratory volume in one second, *FVC* functional vital capacity, *GOLD* the global initiative for chronic obstructive pulmonary disease

^a^Not enough finite observations for GOLD 4

Other than a simple thresholding method for quantitative measurement of air-trapping, some studies aimed at differentiating air-trapping from those remaining in emphysematous regions by the commonly used ratio between the mean lung density at expiration and inspiration (E/I) ratio [16] and more sophisticated method such as parametric response mapping (PRM) [10]. With the fact of LAV% on expiratory CT is the mixture of air-trapping and fraction of emphysema in mind, we studied the correlation between PFT and the mixing effect of LAV%_Emph_ and LAV%_AirT_ by simple addition. The result is shown in the second last category of Table 3. Despite the possible introduction of collinearity, it can be seen that the linearity is strengthened, achieving higher correlation coefficients in the total dataset (*n* = 100). Furthermore, we studied the correlation between PFT and the composite effect of emphysema and functional small airway disease, two main components of COPD. In the last category, LAV%_Emph950+fSAD_ has shown the strengthened correlation analysis as expected for eliminating the overlapped area of emphysema from expiratory CT. These findings affirm the concept that the heterogeneity of airflow limitation (FEV_1_% predicted and FEV_1_/FVC% in this study) is the result of the non-linear contribution from the low attenuation lesions revealed in parenchyma on inspiratory and expiratory CT. Another interesting result showed that Emph% and AirT% have a noticeable stronger correlation with FEV_1_/FVC than FEV_1_% predicted (in agreement with the findings of previous studies [7, 9, 15, 17, 18]). Hence, we use FEV_1_/FVC % as the predicting target in this study.

### Cluster Analysis of LAD

**Fig. 3:**
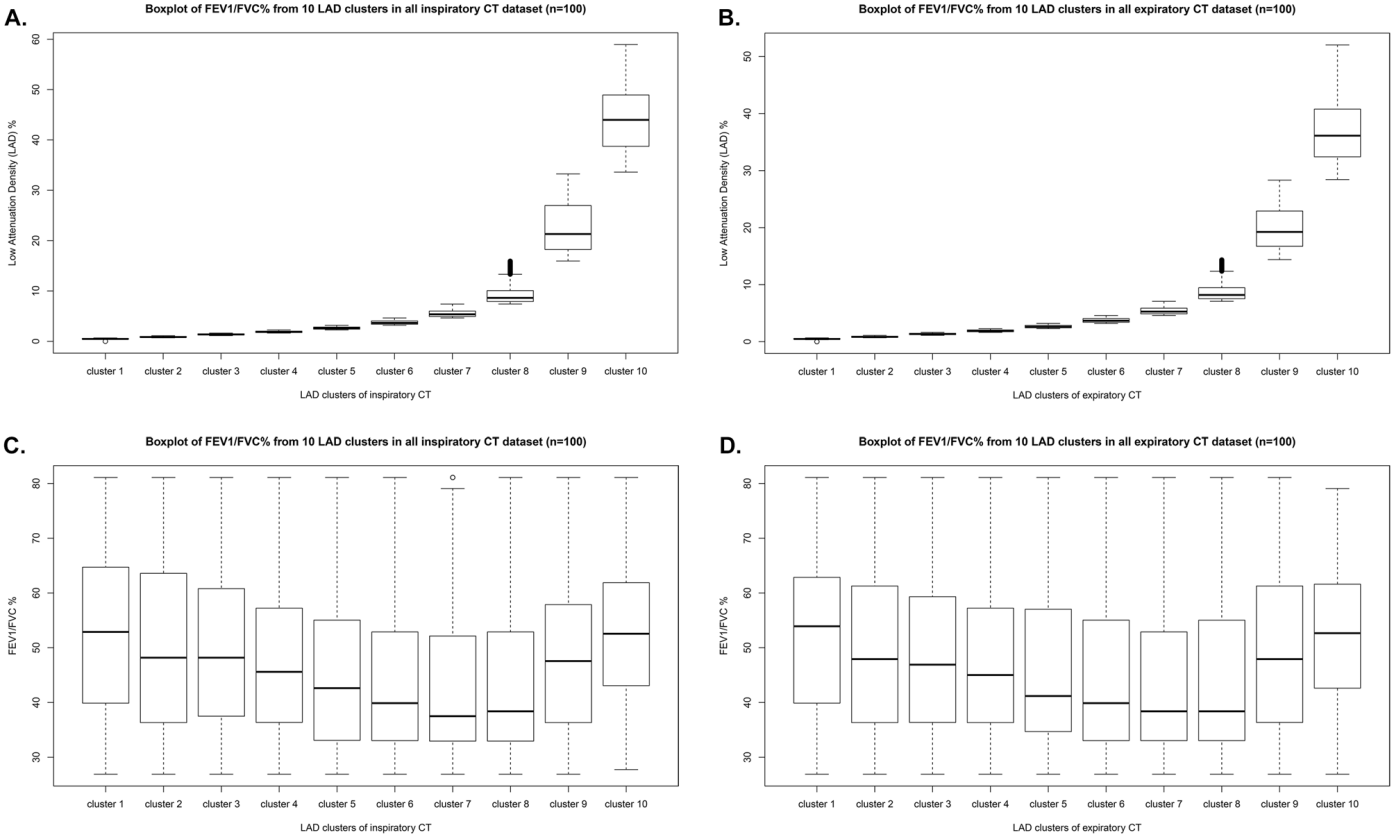
Cluster analysis of low attenuation densities (LADs) on inspiratory CT and expiratory CT. Comparison of 10 scales of LAD% clustered by univariate K-means clustering (inspiratory CT on **A**, expiratory CT on **B**), and comparison of FEV_1_/FVC% distributions of LADs (inspiratory CT on **C** and expiratory CT on **D**)

In the previous study [5], we have developed predictive modeling of FEV1/FVC using LAD distribution on inspiratory CT to predict the functional severities of subjects, differentiated in spirometry but equivalent in LAV%, as well as those equivalents in spirometry but differentiated in LAV%. In this study, we extend the concept to apply the cluster analysis on expiratory CT. Unlike the 4 empirical scales used in the previous study, the LADs were clustered into 10 scales by univariate K-means clustering method. A noticeable similarity in boxplots of 10 scalar clusters is illustrated in Fig. 3 A (inspiratory CT) and B (expiratory CT). Figure 3C, D are the boxplots showing the total FEV1/FVC distribution of each scalar clusters for all the data (*n* = 100). The total number of clusters obtained on inspiratory and expiratory CT is more than 8 million. Although the studied subject can be composed of LADs varied in sizes within lobes, the distribution shown in boxplots in Fig. 3 can be seen as the increased probability of having severe airflow limitation when the heterogenous lung destruction is composed of more LADs in cluster sizes 6 to 8.

### Linear Model Fitting and Derivation of the Predictive Model

It is well understood that the mechanism and transition of how low attenuation relates to disease progression are not yet clear. We hypothesized that the severity of airflow limitation can be partially attributed to the effects of LAD% at a different level according to the size and location of the LAD. To evaluate the effects of the LADs on the severity of lung function, we formulate the full model of multiple linear regression using 10 LAD scales from upper lobes and 10 LAD scales from lower lobes on inspiratory and expiratory CT. A total of 40 LAD scales formulate the predictors used in the full model. Substantial collinearity is expected because of the overlapped effect due to the partial LAC_Emph_ inevitably existing within the LAC_fSAD_ on expiratory CT. Backward selection is applied to improve the statistical significance of the model.

### Prediction Performance Evaluation

In order to evaluate the robustness of the final model, we initially derived the estimated coefficients of linear models on the complete dataset using fivefold cross-validation repeated for 30 times. The Pearson’s correlation between FEV1/FVC% predicted by the model and measured in spirometry is 0.82 (*p* < 0.01) as compared to Pearson’s correlations between FEV_1_/FVC% and low attenuation volume percentage (LAV%_HU-920_) of mild emphysema and LAV%_HU-950_ of severe emphysema are − 0.64 and − 0.55 respectively. Comparing to inspiratory CT, Pearson’s correlations between FEV_1_/FVC% and LAV%_HU-856_ of air-trapping and LAV%_fSAD_ on expiratory CT are − 0.67 and − 0.62, respectively. The new prediction model using both LAD_emph_ and LAD_fSAD_ has shown a better result (*r* = 0.82) than the previous model (*r* = 0.69) in the prior work.

#### BPRM

**Fig. 4:**
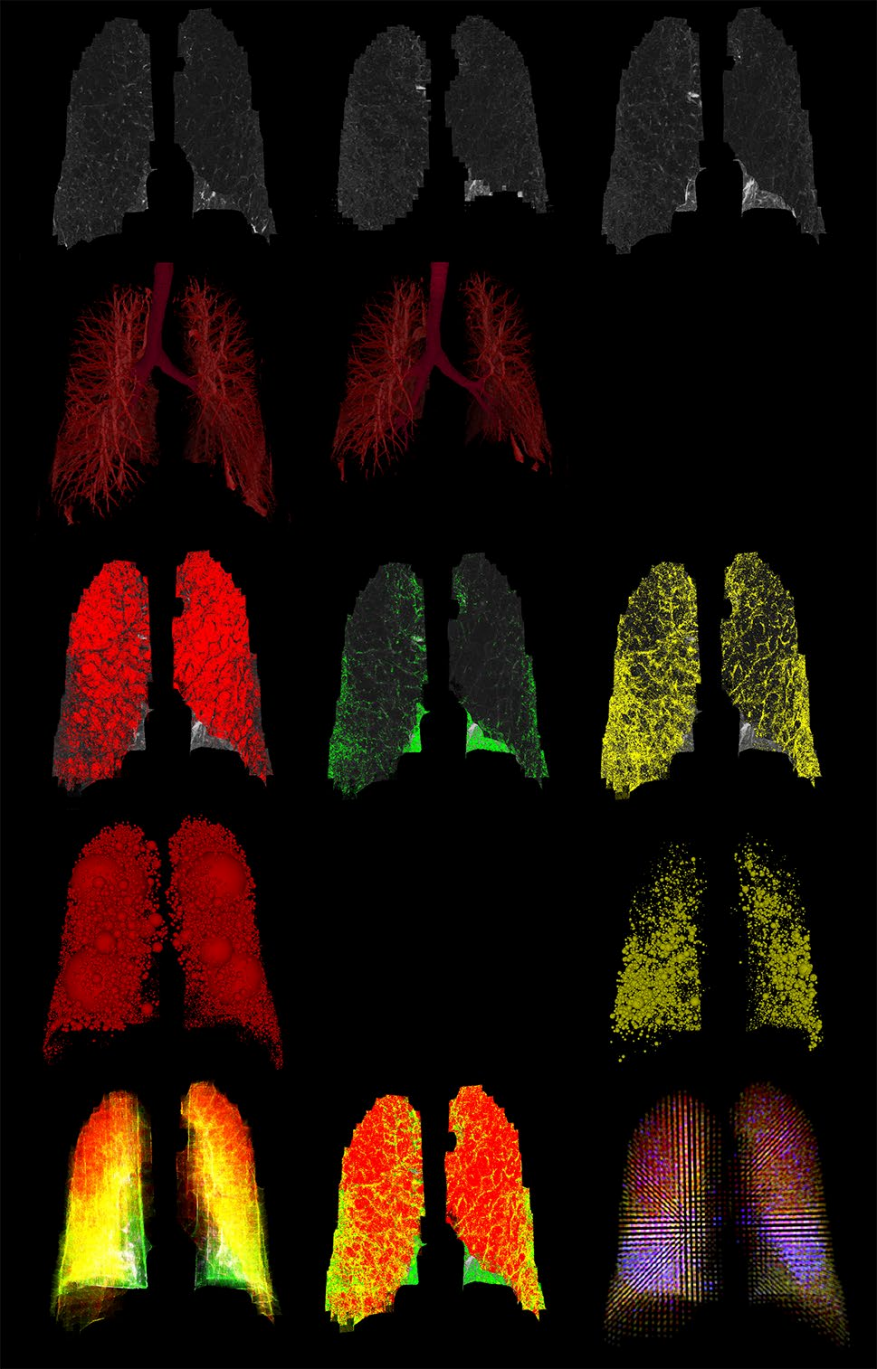
First row: left is inspiratory CT, the center is the original expiratory CT, right is the co-registered result of expiratory CT. Second row: left is the vascular tree rendering of inspiratory CT, and center is the expiratory CT. The visual inspection shows extensive pruning in lower left lobes and relatively mild pruning in lower right lobes. Third row: Red color marks the emphysema regions (HU-950), green color marks the normal regions, yellow marks the air-trapping of expiratory CT, distinguished from emphysema of inspiratory CT using technique of parametric response map (PRM). The fourth row is the result of second-row using low attenuation clusters. Fifth row: left is the visualization result of PRM in a three-dimensional model, the center is the mapping of the PRM on HU values of inspiratory CT, right is the result of regional functional prediction mark on the grids, which has blue color as the most severe candidates for the target lobes

In this study, the regions of the lung are subdivided into grids to illustrate the distribution of functional severity. In contrast to the lobar or whole lung prediction in the prior work, the predicted FEV_1_/FVC% of each subdivision is the summation of each voxel classified by both emphysema LACs on inspiratory CT and the fSAD LACs at the corrected corresponding positions of non-rigid transformed expiratory CT. The bullous PRM (BPRM) is then constructed to provide complementary information when selecting the target segments of BLVR. The result of BPRM is shown in subject 1,003,801 of Fig. 4. This subject has moderate COPD of GOLD 2, FEV_1_% pred: 73.87, FEV_1_/FVC%: 55.03, and the prediction of FEV_1_/FVC% is 50.97, LAV% _fSAD_ 22.76, LAV% _Emph950_ 39.9, LAV% _AirT_ 63.99, and LAV% _Emph920_ 64.38. BPRM indicates that the most functionally destructed lesions are located in the lower lobes where the mixtures of air-trapping and emphysema are mainly located and surrounded by vascular pruning.

## Discussion

**Fig. 5:**
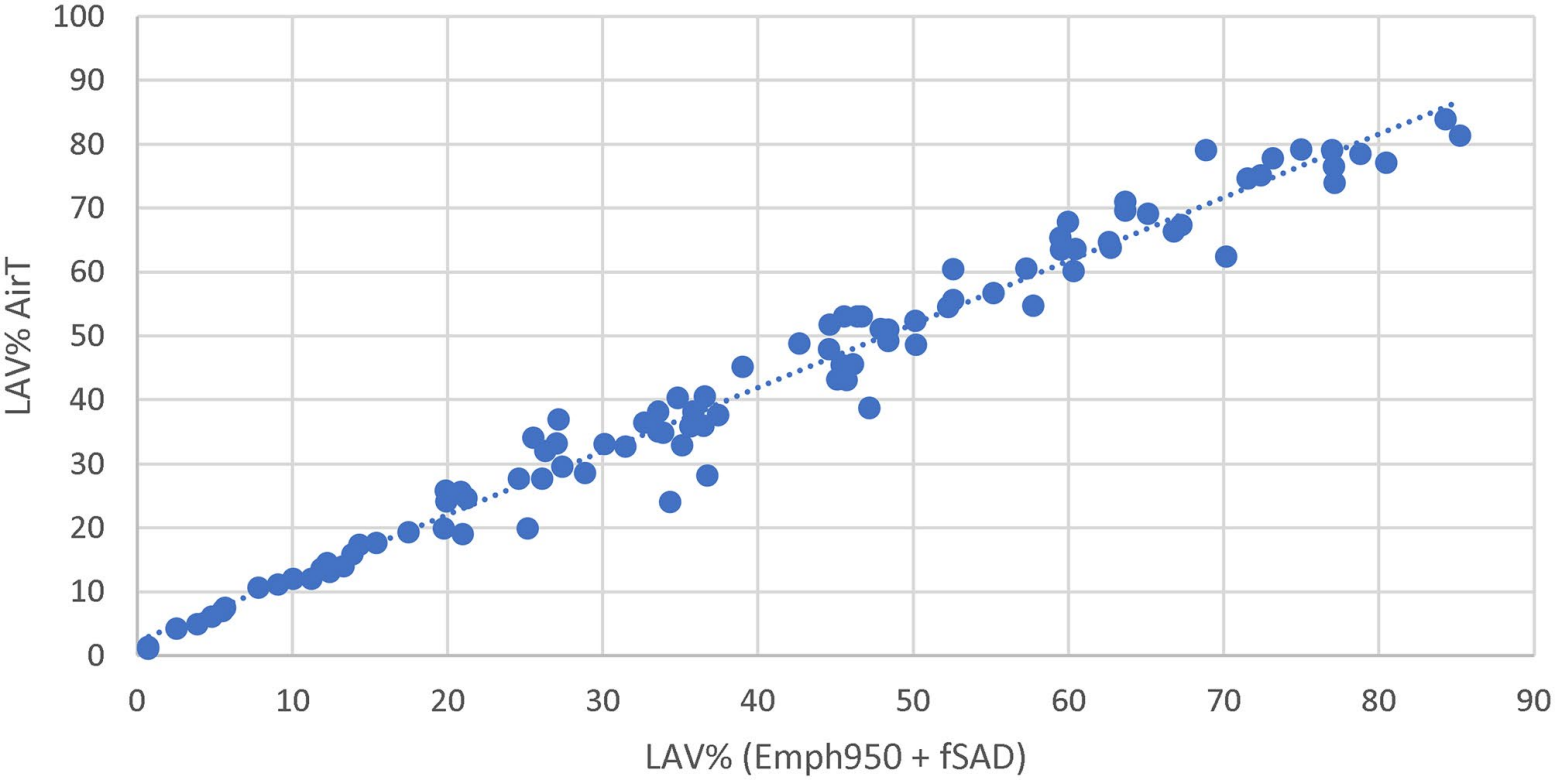
Scatter plot of LAV%_AirT_ using HU-856 threshold on expiratory CT and LAV%_Emph950 + fSAD_ using classification results of PRM. The result has shown a linear relationship in LAV%

In this study, the correlation between LAV%_Emph920_ and PFTs is only statistically significant in the lungs with a severe GOLD stage. The result showed that LAV%_Emph920_ does not have a linear association with PFTs, and LAV%_AirT_ correlates better than LAV%_Emph920_ with PFTs at each GOLD stage. LAV%_AirT_ encloses areas of air-trapping as well as emphysematous regions, which can be decomposed into LAV%_Emph950_ and LAV% _fSAD_ as shown in Fig. 5. Accessing the functional distribution of low attenuation clusters (LACs) in different sizes, we found that giant bullous emphysema (cluster-10) does not necessarily attribute more impact on the lung function when comparing to smaller bullae. Weaker correlation with PFTs is therefore expected in the lungs consisting of giant bullous emphysema. Clinical studies have shown the heterogeneous functional outcome among the subtypes of emphysema. Larger bullae do not necessarily attribute to severe COPD, and smaller, evenly distributed bullae might be more common in the lobe with advanced destructive emphysema.

From the pathophysiological perspective, pursuing a linear relationship between PFTs and LAV% would be more challenging. The centrilobular emphysema (CLE), panlobular emphysema (PLE), and paraseptal emphysema (PSE) are three main phenotypes of emphysematous destruction seen in COPD. PLE refers to diffuse emphysema across the lobule, whereas CLE is considered the primary lesion of destruction at respiratory bronchioles. Not only that each subtype of emphysema differs in sizes, distribution, and location, but they may also have different pathophysiologic contributions to the increase in small airway resistance in COPD. For example, PLE is considered less associated with small airway obstruction than CLE, because the alveolar destruction in PLE is milder than that in CLE, which at the later stage spreads to fuse destroyed lobules and becomes large bullous lesions [19, 20]. Other studies reported that PLE although represents more advanced emphysema has a weaker association with FEV_1_% as compared to CLE [21]. Moreover, previous studies reported the confounding functional effect of bullous emphysema (advanced CLE) in patients with diffuse emphysema (PLE) [22].

Recent studies have shown that the presence of fSAD might be predictive of spirometry decline [18, 23]. From the functional perspective, the extent of hyperinflation can be quantified by the decline in FEV_1_% as the result of increasing bronchitis/bronchiolitis in small airway disease. Detection of LAC_fSAD_ might not only provide detection of COPD at its early stage but also differentiate the functional severity among heterogenous COPD phenotype by the assessment of regional association with PFTs. Another study reported that current smoker with regional air trapping tends to have less emphysema and better lung function than those without [16]. More studies have hypothesized that small airway disease might be a precursor to emphysema [7, 18, 24]. As a result, we extend our previous work of emphysema on inspiratory CT to include fSAD on expiratory CT, in order to provide better functional correlation. Our findings of the better correlation between combined LAV%_Emph950+fSAD_ and FEV1/FVC% support our hypothesis that emphysema and air-trapping are complementary to each other in association with airflow limitation.

The current target lobe selection in BLVR is based on densitometric data on inspiratory CT scans. In the work of Kloth et al. [25, 26], the cluster analysis of LACs representing connected voxels of HU values under -950 was conducted using Pulmo 3D software (Siemens Healthcare). Unlike the fraction of density integrated into this study, Kloth et al. categorized the LACs of inspiratory CT in four empirical volume clusters to visually differentiate the homogeneous and heterogeneous of the emphysema phenotypes. Studying the treatment response of BLVR’s endobronchial coiling, they found that the emphysema phenotypes of the target lobe had no significant impact on the outcome [26]. However, it has to be considered that determining the emphysema heterogeneity in our study is more than qualitative analysis. Lacking the objective image descriptor for emphysema phenotypes with pathophysiological meaning, we pursue the regional COPD severity with the functional prediction from the compositions of the LADs.

The proposed predicting model for airflow limitation using low attenuation data on respiration CT has shown a remarkably strong correlation with statistical significance. Although the emphysematous destruction might be caused by more than one phenotype, making it more difficult to identify the disease trajectory, the longitudinal changes using the proposed model should be studied to provide clinical insight and utilization.

We are aware of the limitations of this retrospective study. We note that the major limitation is the number of subjects included in the study, particularly those at GOLD stage 4. Secondly, the study does not include the measurement of airway wall thickness, although it has been shown to have an association with lung function only in CLE [20]. Another limitation, as well as the future work of this study, is the co-registration of inspiratory and expiratory CT. The transition of LACs in respiration has been studied previously to assess the expansion and collapse of the LACs [10, 27, 28]. Future studies will extend the utility of LACs localization and qualification to distinguish emphysema subtypes.

## Conclusions

In conclusion, the pattern of lung destruction revealed in the low attenuation clusters (LACs) of co-registered CT images has a direct impact on the lung functions. The result of analyzing PFT and LAV% correlation, which is also supported by other studies, shows that LAV%_AirT_ on expiratory CT has a better association than LAV%_Emph_ on inspiratory with spirometry measurements. We have extended the analysis and showed the appreciated correlation contributed by combing both fSAD on expiratory CT and emphysema on inspiratory CT. On top of the PFT correlational analysis, in the categorical analysis of low attenuation volume percentage (LAV%) at a different GOLD stage, we have shown that only patients suffering from severe COPD have a moderate correlation with LAV% on inspiratory CT. The heterogeneity of the disease phenotype is thus evidently affecting the functional outcome measured by PFT. After collecting more than 8 million LACs from 100 subjects, the cluster analysis divides the LACs into 10 scales according to the occupying fractions. The percentage occupying the parenchyma by each LAC can be seen as a fraction of total low attenuation density (LAD). The distribution of lung function in each LAD has shown that the size of LACs has a non-linear relationship with lung function. Particularly, the subjects with medium-sized LACs tend to have weaker lung function than those with diffusive lesions which form into much larger LACs. Furthermore, these LACs are distributed across the entire lung, and their spatial information also has a functional contribution to the disease. Together with the size and the location of LACs, the heterogeneity of emphysema and fSAD can be generalized by the total volume of each LAD category in upper and lower lobes. Utilizing the derived predictive modeling of airflow limitation, we are able to achieve a much stronger correlation between CT image predictors and PFT measurements. The low attenuation density distribution was furtherly incorporated into the BPRM to show a promising result in identifying regional COPD severity for the treatment planning of BLVR.

## Declarations

### Disclaimer

The interpretation and conclusions contained in this study are those of the authors alone. A total of 88 subjects with symptoms of chronic obstructive pulmonary disease (GOLD stage 1 ~ 4) and 12 normal subjects was included; 55 cases of NTUH, and 45 cases of CTH.
